# Intermittent catheterization: A patient-centric approach is key to optimal management of neurogenic lower urinary tract dysfunction

**DOI:** 10.3389/fruro.2023.1158260

**Published:** 2023-03-28

**Authors:** Andrei Krassioukov, Blayne Welk, Desiree Vrijens, Sabrina Islamoska, Kim Bundvig Barken, Veronique Keppenne, Michel Wyndaele, Matthias Walter

**Affiliations:** ^1^ International Collaboration on Repair Discoveries (ICORD), Faculty of Medicine, The University of British Columbia (UBC), Vancouver, BC, Canada; ^2^ GF Strong Rehabilitation Centre, Vancouver Coastal Health Authority, Vancouver, BC, Canada; ^3^ Division of Physical Medicine and Rehabilitation, Faculty of Medicine, The University of British Columbia (UBC), Vancouver, BC, Canada; ^4^ Department of Surgery and Epidemiology & Biostatistics, Western University, London, ON, Canada; ^5^ Department of Urology, Maastricht University Medical Center, Maastricht, Netherlands; ^6^ Evidence & Education Medical Affairs, Coloplast A/S, Humlebaek, Denmark; ^7^ Evidence and Adoption Medical Affairs, Coloplast A/S, Humlebaek, Denmark; ^8^ Department of Urology, Centre Hospitalier Universitaire (CHU) de Liege, Liege, Belgium; ^9^ Department of Urology, University Medical Center Utrecht, Utrecht, Netherlands; ^10^ Department of Urology, University Hospital Basel, University of Basel, Basel, Switzerland

**Keywords:** intermittent catheterization, neurogenic lower urinary tract dysfunction, neurourology, access to health care, patient safety

## Abstract

The value of disposable, single-use catheters has come under scrutiny in recent years with a growing attention on environmental sustainability. Intermittent catheterization (IC) is a widely available and minimally invasive technique for management of lower urinary tract dysfunction. Effective IC for individuals with neurogenic lower urinary tract dysfunction can promote their independence and improve quality of life. Are there alternative options within IC that could minimize environmental impact without compromising the safety and effectiveness of single-use catheters? How does the future of IC look – environmentally friendly, biodegradable, disposable catheters may be complementary to certified reusable catheters? In the midst of this debate, it is important to emphasize that individuals have the right to choose the best evidence-based treatment available. Here we consider the current landscape for IC with a focus on chronic use in individuals with neurogenic lower urinary tract dysfunction.

## 1 Introduction

A considerable number of individuals living with neurogenic lower urinary tract dysfunction (NLUTD) rely on intermittent catheterization (IC) for their bladder management. Currently, there is much debate over which type of catheter – single-use or reusable – provides the safest and most effective management solution, whilst minimizing cost and environmental impact. This article considers the current landscapes for single-use and reusable catheters.

NLUTD is a consequence of various neurological disorders, such as spinal cord injury (SCI), multiple sclerosis (MS), stroke, dementia, spina bifida (SB), and peripheral neuropathy, such as diabetes mellitus (1, 2). The extent of NLUTD depends on the location (e.g., brain region or spinal cord level) and severity of neurological impairment (3). Common symptoms of NLUTD include urinary incontinence, urinary retention, and/or changes in bladder and urethral sensations (3–5).

The overall global prevalence of neurourological disorders, such as NLUTD, is difficult to establish (6). Nevertheless, prevalence rates for the various underlying conditions alongside sporadic data on the frequency of neurourological symptoms in these populations can provide a useful indication of the potential scale of the problem (**Table 1**).

**Table 1:** Epidemiology of neurological conditions underlying urinary tract disorders.

Neurological disorder	Global frequency of disorder	Frequency of urinary tract disorders*	Rate of IC use
Spinal cord injury	13 per 100,000 people (incidence)^†^ (7)	82% of individuals have LUT symptoms or managed NLUTD (8)	45% of individuals with SCI are using IC (9)
Multiple sclerosis	44 per 100,000 people (prevalence) (10)	32–96% of individuals experience NLUTD symptoms depending on disease severity (1)	8% of individuals with MS use IC, if including both past and present use, it is 21% (11)
Stroke	203 per 100,000 people (incidence)^†^ (7)	>50% of individuals report urinary incontinence during the acute phase (1)	Use of intermittent catheters is a viable option for some individuals following stroke in the early rehabilitation phase (12)
Dementias (including Alzheimer’s disease)	712 per 100,000 people (prevalence)^†^ (7)	11–93% of individuals with dementia experience urinary incontinence (13, 14)	Cognitive dysfunction is a major barrier to learning IC (15). Number of individuals using intermittent self-catheterization or assisted IC is unknown
Spina bifida	33.86 per 100,000 live births (prevalence)^‡^ (16)	>90% experience NLUTD (1)	89–100% of individuals with spina bifida use IC (17)
Diabetes mellitus	10.5% of adult population^§^ (18)	62% of individuals have NLUTD^¶^ (19)	Diabetic cystopathy typically appears when the disease is in its advanced stage (19), for those who cannot empty their bladder, IC is recommended (20)

*Data are from heterogeneous sources and, therefore, may vary across different geographical locations. ^†^Age-standardized rate (2016 data). ^‡^Data from geographical regions where folic acid fortification was mandatory at the time of data collection. ^§^Aged 20–79 years. ^¶^NLUTD caused by diabetic peripheral neuropathy.

IC, intermittent catheterization; LUT, lower urinary tract; MS, multiple sclerosis; NLUTD, neurogenic lower urinary tract dysfunction; SCI, spinal cord injury.

The Global Burden of Disease study (2016 data) has shown that neurological disorders are the leading cause of disease burden on a global scale, with stroke being the largest contributing factor (7, 21). With an aging population and an increase in the number of individuals affected by neurological disorders, this global burden continues to grow, posing a challenge to healthcare systems, many of which are already overstretched (7). In addition, the added burden of NLUTD in individuals with neurological disorders places even more pressure on healthcare systems.

NLUTD has been shown to have a significant socio-economic burden (22), while also having a considerable negative impact on an individual’s health-related quality of life (HRQoL) (23–27). A Danish registry study has demonstrated a significantly greater number of total hospitalizations, outpatient visits and primary healthcare contacts, and longer inpatient stays during the first year after a diagnosis of NLUTD in individuals with SCI or MS versus controls from the general population (matched by age, gender, marital status, municipality, and education; all p<0.05) (5). The burden specifically due to lower urinary tract (LUT) and bowel complications was also significantly increased (5). These complications often required hospitalization and antibiotic therapy (5, 28). Furthermore, earned income was reduced by up to threefold versus matched controls, which was countered by an increase in income transfer payments, mainly in the form of disability pension and sick pay (5).

Neurological disorders and associated NLUTD can restrict many aspects of an affected individual’s life (29). For example, individuals with MS, SCI, or stroke who are experiencing NLUTD compared with neurologically intact individuals with normal independent bladder function, reported poorer HRQoL in terms of their physical, mental, and sexual health, and in other important aspects of life, such as socializing and travel (24). Improvements in HRQoL in these individuals can be achieved through effective management of NLUTD, with the goals of continence, voluntary bladder emptying (often with IC), preserved renal function, and a low risk of urinary tract infections (UTIs) (6, 30, 31). If not addressed, UTIs can escalate to urosepsis, a severe medical condition that can be life-threatening and can have considerable negative impacts on individuals and healthcare (32, 33). Crucially, for many individuals with NLUTD, effective management helps them to retain an element of independence in their everyday lives (34).

Approaches to the management of NLUTD vary between clinical settings and there is no evidence that any one particular solution is optimal for all (35). An appropriate intervention should be carefully considered and aligned with the individual’s profile (e.g., extent of NLUTD, potential previous LUT surgery, anatomical factors, dexterity, and general motor/cognitive ability) and personal preferences to promote the best chances of effective management for NLUTD (35). Effective management of NLUTD must be supported by appropriate training from multi-disciplinary teams (36).

## 2 Clean intermittent catheterization as standard of care for the management of NLUTD

Clinical guidelines recommend IC as the gold standard treatment for individuals with NLUTD with sufficient dexterity, who are unable to empty their bladder (6, 35, 37). IC is a widely available and minimally invasive technique that aims to promote an individual’s independence and improve their HRQoL (38).

Since the 1970s, IC has been performed using a ‘clean’ technique, which involves handwashing, regular genital hygiene, and catheter cleaning before reuse (35, 38). Over the years, the practice of IC has evolved from reusable to single-use catheters (38), due, in part, to a lack of evidence on appropriate storage and cleaning procedures, a perceived increased risk of UTIs for reusable catheters (39, 40), and a lack of certified and available reusable catheters in many countries. Currently, there is no conclusive evidence that reusable catheters are as safe as single-use (41). Longitudinal studies are necessary to establish the efficacy and safety profile of cleaning procedures for reusable catheters (42). Given the complexity of underlying conditions of individuals who require management for NLUTD, there is no single type of IC that is suitable for all. Based on the current evidence available, hydrophilic single-use catheters are considered to be the optimal choice for management of NLUTD due to a reduced risk of UTIs, improved HRQoL, and individual preference (31, 35, 40, 43–45). Individual preference is likely to improve compliance with IC which, in turn, ensures long-term successful management of NLUTD (44). However, depending on geographical location, access to specific types of catheters is limited by reimbursement policies, under-resourcing, and issues with funding (39). In the US, restrictions on the type of catheter available and on the quantities provided to users are important factors challenging the health and well-being of individuals with NLUTD (46). Many private insurers do not provide enough catheters per month to cover the number of catheterizations needed each day and, consequently, people resort to reusing single-use catheters, even though this is considered ‘off-label’ use (38). In Europe, only disposable single-use catheters are reimbursed (44, 47, 48). The situation is very different in developing countries where resources and funding are limited, which means that reusable or reuse of disposable catheters may be the only option available (3, 39).

Recently published reports highlight a growing concern over the environmental impact of nonbiodegradable plastic waste resulting from the use of disposable, single-use catheters (40, 47, 49), including the products, packaging, manufacturing processes, and transportation. It has been suggested that this environmental impact may be alleviated by using reusable catheters (40, 47, 49). Reusable catheters have also been advocated as a means of reducing the cost of managing NLUTD (41). There have been proposals that reusable catheters are more cost-effective than single-use options (41, 50); however, the available economic data do not consider the influence of individual preference for type of catheter, the individual variation in the daily frequency of catheterizations, the environmental and financial impact of catheter cleaning protocols, or the impact of downstream complications, such as UTIs. In countries where the cost can be covered by the healthcare system or individuals, single-use catheters should be considered as the preferred routine method of choice for the management of NLUTD (39).

## 3 Reusable catheters as a solution for the management of NLUTD

In an era where sustainability is a key focus for developed countries, and limited funding/access to healthcare resources remains a problem for developing countries, the value of disposable, single-use catheters is firmly under the spotlight (51). Arguably, reusable catheters alleviate the environmental and economic pressures of catheterization, whereas single-use catheters address user comfort and the risk of UTIs (52); all of which are important considerations for individuals using IC (40). Presently the vast majority of IC are produced with single-use indication.

Reusing catheters requires appropriate, effective cleaning to reduce the risk of bacteria entering the bladder (53), and to avoid damaging the structural integrity of the catheter (42). Various catheter cleaning methods have been evaluated but, as yet, no uniform clinical recommendations on how to clean and reuse catheters exist (42, 44, 54). Evidence on the safety and efficacy of different cleaning methods must be established to help guide future clinical guidelines (42). Furthermore, the cleaning processes involved in using reusable catheters may not be achievable for all individuals with NLUTD, for example, individuals with cognitive impairment, restricted hand dexterity, or limited access to clean water, and may represent an additional burden for caregivers. Importantly, ‘off-label’ reuse of catheters that are intended and approved for single-use can, potentially, lead to unsafe practice, health risks, and other complications (55). It would also be prudent to consider the environmental impact of the cleaning fluids used in catheter reuse processes.

Whether the incidence of UTIs is affected by the type of catheter remains a matter of debate. Some studies suggest that the use of hydrophilic-coated catheters considerably lowers the risk of UTIs versus other catheters (31, 56, 57). Hydrophilic-coated catheters have also been reported to be cost-effective compared with uncoated catheters (58, 59). In contrast, a retrospective study of individuals with SCI in the US found that a shift from reusable to single-use catheters did not decrease hospitalizations for genitourinary symptoms, including UTIs (60). To date, however, no randomized controlled trial of sufficient sample size and adhering to an up-to-date UTI definition including catheter-associated UTIs has been conducted to evaluate the effectiveness of reusable versus single-use catheters (48, 61). The COMPaRE study (Netherlands) and the Mult*IC*ath study (UK) are ongoing large-scale clinical trials designed, primarily, to determine if the incidence of UTIs is the same between reusable and single-use catheters (48, 62). A recent publication by Welk et al., 2022 reported a significantly higher risk of UTIs in individuals with neurogenic versus non-neurogenic conditions using IC (63). Therefore, considering the presence of an underlying neurological disorder in a mixed population of individuals with neurogenic and non-neurogenic conditions is key when interpreting data, as the treatment needs of these groups differ based on the underlying condition and its severity, course of disease, and IC dependency.

To consider the use of reusable catheters as a routine clinical practice, an appropriate framework for management, supported by evidence from adequate randomized clinical trials, which considers the individual’s disability and cognitive status, alongside any potential risks, must be available. In addition, training and educational resources for healthcare professionals should be implemented to ensure that individuals receive the support they require for effective management of NLUTD. Until there is scientific evidence that reusable catheters are not inferior to single-use with respect to long-term complications as mentioned previously, healthcare providers (HCPs) should continue to take a patient-centric approach to catheterization; choosing single-use or reusable catheters (once certified reusable catheters are available), or a combination of both, considering the specific requirements of each individual and their preferences for management of NLUTD. Different groups may require different recommendations, but they all require a simple and easy solution that is not associated with any major risks (safety or otherwise). It is the role of the HCP to ensure that individuals are well-informed of the benefits and risks before starting IC. Furthermore, the choice of catheter should not be based on financial aspects at the detriment of safety.

On the issue of sustainability, the responsibility of ensuring the safety of individuals using catheters (or any other medical device) for treatment purposes must be prioritized over minimizing environmental impact, without disregarding it. All medical devices should be optimally designed to deliver best performance on their intended purpose and manufactured using appropriate high-quality materials. This does not mean that sustainability should be compromised, but instead it should be considered at all steps in the process to achieve the same optimal outcome with the least environmental impact. It is important to consider ways in which the ‘catheter footprint’ could be reduced, for example, recycling used catheters or using biodegradable materials in catheter production (49). There have already been environmentally conscious moves toward reducing bulky packaging and manufacturing packaging from biodegradable and/or recyclable materials. Furthermore, reducing the need for additional lubricants could avoid the associated extra packaging waste. At present, catheters are treated as hazardous waste and have been excluded from recycling consideration (49). Therefore, the creation of facilities equipped to safely recycle used catheters would reduce the waste burden associated with IC (49). There have also been developments in the manufacturing of biodegradable catheters from materials such as water-soluble corn starch-based bioplastic and polyolefin-based elastomer, which have demonstrated better environmental performance compared with standard plastics used in catheter manufacturing (64, 65). It may be more prudent to explore environmentally friendly solutions rather than stopping the use of disposable catheters altogether. These solutions will not only require advances in technology but also changes in current legislation on medical devices to encompass sustainability as well.

## 4 Individuals’ rights

Personal choice and access to the best care are important considerations for individuals when choosing treatment options for IC. Hydrophilic single-use catheters are, currently, regarded as the optimal choice for draining an individual’s bladder, whereas concerns remain over appropriate cleaning methods and infection risk for reusable catheters. Furthermore, from an individual perspective, switching from reusable catheters to single-use hydrophilic-coated catheters improved HRQoL and were the preferred choice among users (31). Acknowledging that sustainability is high on the current global agenda, the plastic waste (and carbon footprint) associated with disposable catheters is a critical issue. Therefore, individuals using plastic-based catheters may feel stigmatized. HCPs must be astute to the possibility of stigma to avoid consequences for mental health, which may already be impaired due to their LUT problems and associated symptom-driven behavior (e.g., frequent trips to the bathroom) (66, 67). Ultimately, HCPs are responsible for informing individuals of the most appropriate IC solution tailored to their individual needs and preferences to promote adherence.

Whilst the sustainability debate continues, it is important to remember that individuals must always have the right to choose the best evidence-based treatment available.

## Data availability statement

The original contributions presented in the study are included in the article/supplementary material. Further inquiries can be directed to the corresponding author.

## Author contributions

All authors listed have made a substantial, direct, and intellectual contribution to the work and approved it for publication.
